# Industry 5.0 concepts and enabling technologies, towards an enhanced conservation practice: systematic literature review protocol

**DOI:** 10.12688/openreseurope.17505.1

**Published:** 2024-04-18

**Authors:** Alejandro Jiménez Rios, Margarita L. Petrou, Rafael Ramirez, Vagelis Plevris, Maria Nogal

**Affiliations:** 1 Department of Built Environment, Oslo Metropolitan University, Oslo, 0130, Norway; 2 Department of Civil and Environmental Engineering, University of Cyprus, Nicosia, 1678, Cyprus; 3 Department of Civil Engineering, University of Minho, Guimarães, 4800-058, Portugal; 4 Department of Civil and Environmental Engineering, University of Qatar, Doha, 2713, Qatar; 5 Materials, Mechanics, Management and Design Department, Delft University of Technology, Delft, 2628, The Netherlands

**Keywords:** Industry 5.0, Human-Centrism, Resilience, Sustainability, Enabling Technologies, Built Cultural Heritage Environment, Conservation

## Abstract

Industry 4.0 has led to digitalization and an increase in industrial activity. However, it has recently been recognized as inadequate for achieving European goals by 2030. Therefore, a novel Industry 5.0 paradigm has emerged in response to the unexpected negative effects caused by its predecessor. Industry 5.0 is mainly based on three foundational ideas: i) human-centrism, ii) resilience, and iii) sustainability. Human-centric solutions and human-machine-interaction; bio-inspired technologies and smart materials; real time-based digital twins and simulation; cyber safe data transmission, storage, and analysis; artificial intelligence; and energy efficiency and trustworthy autonomy have been recognized as the enabling technologies of this transformative vision. This paper outlines the protocol adopted to conduct a systematic literature review with the aim of exploring how the Architecture, Engineering, Construction, Management, Operation, and Conservation (AECMO&C) industry can adapt and be better prepared to embrace novel Industry 5.0 principles and enabling technologies, ultimately resulting in enhanced conservation practices for the built cultural heritage environment.

**Registration:**

The protocol has been registered on Open Science Framework (24/02/2024) and follows the PRISMA-P guidelines.

## Plain language summary

The arrival of "Industry 4.0" has brought a lot of changes to the way industries work, making them more digital. However, it hasn't been enough to meet Europe’s targets for 2030. As a result, a new concept called "Industry 5.0" has been created to fix some of the problems caused by Industry 4.0.

Industry 5.0 is based on three main ideas. First, it focuses on people and how they interact with machines. Second, it aims to create systems that can recover from disruptions. Finally, it emphasizes the need to protect our environment while creating economic and social benefits.

This new concept makes use of different technologies. These include solutions that focus on people and their interaction with machines, technologies inspired by nature, smart materials, virtual copies of physical systems that work in real time, secure data handling, artificial intelligence, and energy-saving measures.

This paper outlines the method used to review a bunch of studies on how the industries of architecture, construction, engineering, management, operation, and conservation can adapt to Industry 5.0. The goal is to help these industries better preserve our cultural heritage buildings. The method used for this review has been officially registered and follows a set of guidelines called the PRISMA-P.

## Introduction

Built cultural heritage refers to man-made structures, buildings, landmarks, and spaces with historical, architectural, artistic, cultural, or social significance ^1^. The United Nations Educational, Scientific and Cultural Organization (UNESCO) promotes worldwide cultural equality and heritage conservation. By adopting the World Heritage Convention (WHC) in 1972, Member States of UNESCO are responsible for identifying, protecting, conserving, and presenting the world’s heritage ^2^. Unfortunately, these invaluable assets are vulnerable to different socio-economic and natural hazards, especially in the face of climate change and its effects ^3^. The United Nations (UN) recognizes in Target 11.4 of the Sustainability Development Goals (SDG) that safeguarding cultural heritage contributes to making cities and human settlements inclusive, safe, resilient, and sustainable ^4^. Thus, endeavors to preserve the built cultural heritage environment are both required and desired ^5^.

Previous work indicates that a suitable Industry 4.0 digital twin framework for built cultural heritage assets would be primarily based on an As-Is Historical Asset Information Model (AI-HAIM) ^6^. This AI-HAIM must contain interoperable data, geometry, finite element, and data-driven modules (“digital asset”) and be linked to its real-world counterpart through a multi-metric asset health monitoring system. That system should continuously produce data on the asset’s structural, environmental, and operational conditions (“physical asset”). Major challenges in creating Industry 4.0 digital twins include: i) lack of interoperability among different software used in the digital twin model generation; ii) unclear guidelines for creating macro-digital twins integrating individual asset models, and iii) absence of Findable, Accessible, Interoperable, and Reusable (FAIR) benchmark databases suitable for digital twin prototyping development and validation ^7, 8^.

While the vision of Industry 4.0 has driven digitalization and increased productivity, it has been deemed inadequate to achieve European goals by 2030 ^9^. Thus, a novel Industry 5.0 paradigm has emerged. Industry 5.0 is mainly based on three foundational ideas: i) human-centrism, ii) resilience, and iii) sustainability. The recognized enabling technologies of this new transformative vision include: a) Individualized human-machine-interaction, b) bio-inspired technologies and smart materials, c) digital twins and simulation, d) data transmission, storage, and analysis technologies, e) artificial intelligence, and f) technologies for efficiency, renewables, storage, and autonomy ^10^.

The objective of this systematic literature review is to collect and synthesize state-of-the-art knowledge and information, identify how Industry 5.0 concepts and enabling technologies can contribute to the improvement of built cultural heritage conservation practices, identify current gaps, and highlight future potential developments and opportunities in the field. To this end, the proposed systematic review will answer the following question: How can the architecture, engineering, construction, management, operation, and conservation (AECMO&C) industry adapt and be better prepared to embrace novel Industry 5.0 concepts ultimately leading to enhanced practices in built cultural heritage conservation?.

## Protocol

While the PRISMA 2020 ^11^ statement was initially developed for use in medical and clinical sciences, its principles can also be applied within the field of engineering. This is because PRISMA 2020 offers a useful guide on how to find, choose, evaluate, and combine information from existing literature. This paper presents the protocol adopted to perform a systematic literature review within the context of an International Centre for the Study of the Preservation and Restoration of Cultural Property (ICCROM) Fellowship, part of the Digital Twin Anomaly Detection Decision-Making for Bridge Management (DTADD) Marie Skłodowska-Curie Actions (MSCA) Individual Fellowship (IF) Postdoctoral Research Project. The protocol has been registered in the OSF Registries website ^12^ and follows the checklist provided by PRISMA-P ^13^ and guidelines of the PRISMA-P Explanation and Elaboration ^14^.

### Systematic literature review administrative information

The title of this protocol, as well as the authors contact, have been provided in the cover page of this manuscript, being the corresponding author also the guarantor. This protocol has been registered in the OSF Registries website under the Generalized Systematic Review Registration Form ^15^ on 24/02/2024 ^12^. In the event of protocol amendments, the date of each amendment will be accompanied by a description of the change and the rationale. This project has received funding from the European Union’s Horizon 2020 research and innovation programme under the Marie Sklodowska-Curie grant agreement No. 101066739. Moreover, support was also provided by the International Centre for the Study of the Preservation and Restoration of Cultural Property (ICCROM) by granting access to ICCROM extensive bibliographic resources during the time this work was conducted to the corresponding author of this paper within the context of a prestigious ICCROM Fellowship. Finally, the European Union funded this research. Nevertheless, the funder is not involved in any other aspect of the project and will have no input on the interpretation or publication of the study results.

### Systematic literature review methods

Different criteria were considered to decide whether a record would be eligible. Peer reviewed records published as Articles, Conference Paper, Book Chapters, or Review in Journals, Conference Proceedings, Books, or Book Series within the subject areas of Computer Science, Engineering, Business Management and Accounting, Decision Sciences, Social Sciences, Economics Econometrics and Finance, Energy, Environmental Science, Materials Science, Arts and Humanities, and Multidisciplinary, from 2020 up to 2024, written in English, and containing the following keywords of interest (and similar terms, namely: human-centrism and human-centric, etc.) as part of the record’s title, abstract, or keywords, will be initially included:

- Industry 5.0.
- Principles:
  ○ Human-Centrism.
  ○ Resilience.
  ○ Sustainability.
- Enabling Technologies:
  ○ Human-centric solutions.
  ○ Human-machine-interaction.
  ○ Bio-inspired technologies.
  ○ Smart materials.
  ○ Digital twins.
  ○ Cyber safe data.
  ○ Artificial intelligence.
  ○ Energy efficiency.
  ○ Trustworthy autonomy.

In combination with:

- Built Cultural Heritage Environment.
- Conservation.

Scopus was the selected electronic database to be searched as it contains comprehensive records on the field of interest and the necessary features to extract bibliographic data. It is foreseen that in case a relatively small number (<20) of records is finally included, both forward and backward citation searching will be conducted to increase this number with relevant records. Furthermore, in case it is required to further increase the number of records, additional records will be searched throughout the extensive bibliographic resources of ICCROM. Three different search queries were run implementing the specified eligibility criteria and combining the various keywords of interest identified. The full search queries were:

- TITLE-ABS-KEY ( "industry 5.0" AND (heritage OR conservation) ) AND PUBYEAR > 2019 AND PUBYEAR < 2025 AND ( LIMIT-TO ( SRCTYPE , "j" ) OR LIMIT-TO ( SRCTYPE , "p" ) OR LIMIT-TO ( SRCTYPE , "k" ) OR LIMIT-TO ( SRCTYPE , "b" ) ) AND ( LIMIT-TO ( DOCTYPE , "ar" ) OR LIMIT-TO ( DOCTYPE , "cp" ) OR LIMIT-TO ( DOCTYPE , "ch" ) OR LIMIT-TO ( DOCTYPE , "re" ) ) AND ( LIMIT-TO ( SUBJAREA , "COMP" ) OR LIMIT-TO ( SUBJAREA , "ENGI" ) OR LIMIT-TO ( SUBJAREA , "BUSI" ) OR LIMIT-TO ( SUBJAREA , "DECI" ) OR LIMIT-TO ( SUBJAREA , "SOCI" ) OR LIMIT-TO ( SUBJAREA , "ECON" ) OR LIMIT-TO ( SUBJAREA , "ENER" ) OR LIMIT-TO ( SUBJAREA , "ENVI" ) OR LIMIT-TO ( SUBJAREA , "MATE" ) OR LIMIT-TO ( SUBJAREA , "ARTS" ) OR LIMIT-TO ( SUBJAREA , "MULT" ) ) AND ( LIMIT-TO ( LANGUAGE , "English" ) )
- TITLE-ABS-KEY ( ( human-centr* OR resilien* OR sustainab* ) AND ( "cultural heritage" AND conservation ) ) AND PUBYEAR > 2019 AND PUBYEAR < 2025 AND ( LIMIT-TO ( SRCTYPE , "j" ) OR LIMIT-TO ( SRCTYPE , "p" ) OR LIMIT-TO ( SRCTYPE , "k" ) OR LIMIT-TO ( SRCTYPE , "b" ) ) AND ( LIMIT-TO ( DOCTYPE , "ar" ) OR LIMIT-TO ( DOCTYPE , "cp" ) OR LIMIT-TO ( DOCTYPE , "ch" ) OR LIMIT-TO ( DOCTYPE , "re" ) ) AND ( LIMIT-TO ( SUBJAREA , "COMP" ) OR LIMIT-TO ( SUBJAREA , "ENGI" ) OR LIMIT-TO ( SUBJAREA , "BUSI" ) OR LIMIT-TO ( SUBJAREA , "DECI" ) OR LIMIT-TO ( SUBJAREA , "SOCI" ) OR LIMIT-TO ( SUBJAREA , "ECON" ) OR LIMIT-TO ( SUBJAREA , "ENER" ) OR LIMIT-TO ( SUBJAREA , "ENVI" ) OR LIMIT-TO ( SUBJAREA , "MATE" ) OR LIMIT-TO ( SUBJAREA , "ARTS" ) OR LIMIT-TO ( SUBJAREA , "MULT" ) ) AND ( LIMIT-TO ( LANGUAGE , "English" ) )
- TITLE-ABS-KEY ( ( human-centr* OR human-machin* OR bio-inspired OR "smart material*" OR "digital twin*" OR "cyber safe" OR "artificial intelligence" OR "energy efficien*" OR "trustworthy autonom*" ) AND ( "cultural heritage" AND conservation ) ) AND PUBYEAR > 2019 AND PUBYEAR < 2025 AND ( LIMIT-TO ( SRCTYPE , "j" ) OR LIMIT-TO ( SRCTYPE , "p" ) OR LIMIT-TO ( SRCTYPE , "k" ) OR LIMIT-TO ( SRCTYPE , "b" ) ) AND ( LIMIT-TO ( DOCTYPE , "ar" ) OR LIMIT-TO ( DOCTYPE , "cp" ) OR LIMIT-TO ( DOCTYPE , "ch" ) OR LIMIT-TO ( DOCTYPE , "re" ) ) AND ( LIMIT-TO ( SUBJAREA , "COMP" ) OR LIMIT-TO ( SUBJAREA , "ENGI" ) OR LIMIT-TO ( SUBJAREA , "BUSI" ) OR LIMIT-TO ( SUBJAREA , "DECI" ) OR LIMIT-TO ( SUBJAREA , "SOCI" ) OR LIMIT-TO ( SUBJAREA , "ECON" ) OR LIMIT-TO ( SUBJAREA , "ENER" ) OR LIMIT-TO ( SUBJAREA , "ENVI" ) OR LIMIT-TO ( SUBJAREA , "MATE" ) OR LIMIT-TO ( SUBJAREA , "ARTS" ) OR LIMIT-TO ( SUBJAREA , "MULT" ) ) AND ( LIMIT-TO ( LANGUAGE , "English" ) )

As can be observed from the reported queries, the search was limited to the search fields of title, abstract and keywords. Moreover, a series of filters were applied, namely: records published within the years 2020 and2024, document types pertaining to the categories of articles, conference papers, book chapters, or reviews, published in sources such as journals, conference proceedings, books, or book series, written in English, and within the subject areas of computer science, engineering, business management and accounting, decision sciences, social sciences, economics econometrics and finance, energy, environmental science, materials science, arts and humanities, and multidisciplinary.

The search strategy adopted has been created specifically for this systematic review, thus, no search strategies from previews literature reviews have been adopted. Furthermore, no update method will be used (although Scopus allows the use of search alerts) as the final manuscript will be completed shortly after the search had been performed. The search was conducted on 16/02/2024. Full details of the search strategy conducted have been published online and are openly available for the reviewers of this protocol manuscript, and anyone else interested on consulting them ^16, 17^.

The total records found will be reported in accordance to the PRISMA 2020 flow diagram for systematic reviews ^11^. The number of records identified with each of the search queries reported were: SQ1 = 13, SQ2 = 777, SQ3 = 117. A FAIR bibliographic database has been created with all the records identified at this stage ^18^. The deduplication process was conducted automatically through the EndNote software. The number of records for each one of the search queries after deduplications were: SQ1 = 13, SQ2 = 776, SQ3 = 83.

Both a bibliometric analysis and a narrative synthesis will be performed to qualitatively present the results of the systematic literature review. The information of the included records will be qualitatively summarized in a narrative synthesis as the findings are characterized by heterogeneity. Moreover, two approaches for the bibliometric analysis will be conducted: a performance analysis and a science mapping. The performance analysis will be presented in terms of publications per year, most cited authors, most cited records, documents per country, keyword occurrence, and most used source for publication. On the other hand, the science mapping will analyze the co-authorship relationships in terms of authors and countries, as well as the co-occurrence relationships between keywords (both author and index keywords). No meta-bias assessment is planned for the proposed systematic review and no confidence assessment plan will be used for the proposed systematic review. This is based in the fact that no universally accepted methodology is available for the grading of reviewing in the AECMO&C field.

## Conclusions/Discussion

In conclusion, the Industry 5.0 paradigm, with its focus on human-centrism, resilience, and sustainability, offers a transformative vision for the AECMO&C industry. This paper has developed a protocol for a systematic literature review to explore how this industry can adapt to and incorporate the principles and enabling technologies of Industry 5.0. These technologies include human-centric solutions and human-machine interaction, bio-inspired technologies and smart materials, real time-based digital twins and simulation, secure data transmission, storage, and analysis, artificial intelligence, and energy efficient and trustworthy autonomy. The goal is to improve conservation practices for the built cultural heritage environment. The protocol, which follows the PRISMA-P guidelines, is registered and ready for implementation. This research aims at providing a roadmap for the AECMO&C industry to navigate and thrive in the upcoming Industry 5.0 landscape.

## Ethics and consent

Ethical approval and consent were not required.

## Data Availability

Zenodo: Bibliographic Dataset for the Systematic Literature Review on Industry 5.0 Concepts and Enabling Technologies, Towards an Enhanced Conservation Practice. https://zenodo.org/doi/10.5281/zenodo.10671411 ^18^ Zenodo: Industry 5.0 Concepts and Enabling Technologies, Towards an Enhanced Conservation Practice: Systematic Literature Review Search Strategy Checklist. https://zenodo.org/doi/10.5281/zenodo.10875793 ^16^ Zenodo: PRISMA-P checklist for ‘Industry 5.0 concepts and enabling technologies, towards an enhanced conservation practice: systematic literature review protocol’. https://zenodo.org/doi/10.5281/zenodo.10877563 ^17^ Data are available under the terms of the Creative Commons Attribution 4.0 International license (CC-BY 4.0).
