# Supplementary material for: Industry 5.0 concepts and enabling technologies, towards an enhanced conservation practice: systematic literature review protocol
Source: Open Res Eur. 2024 Apr 18;4:75. [Version 1] doi: 10.12688/openreseurope.17505.1 (PMC11255542; doi:10.12688/openreseurope.17505.1)
Supplement: Peer review history

# Reviewer report: Reviewer response for version 1

**DOI:** 10.21956/openreseurope.18919.r41895

**Published:** 2024-07-17

**Authors:** James Hutson

**Affiliations:** 1 Lindenwood University, Saint Charles, Missouri, USA

The article presents a protocol for a systematic literature review aimed at exploring how Industry 5.0 concepts and enabling technologies can enhance conservation practices within the Architecture, Engineering, Construction, Management, Operation, and Conservation (AECMO&C) industry. The Industry 5.0 paradigm emphasizes human-centrism, resilience, and sustainability, addressing the limitations of Industry 4.0 in achieving European goals for 2030. The study seeks to synthesize existing knowledge, identify gaps, and propose future directions for integrating Industry 5.0 technologies in the conservation of built cultural heritage.

 The rationale for, and objectives of, the study are clearly described. The article outlines the necessity to transition from Industry 4.0 to Industry 5.0 due to the former’s inadequacies in meeting European targets. It highlights the emergent need for human-centrism, resilience, and sustainability within industrial practices. The objectives are explicitly stated, focusing on synthesizing state-of-the-art knowledge, identifying gaps, and proposing future developments in the field. The rationale is well-justified, presenting a compelling case for the relevance and urgency of the study.

 The study design is appropriate for the research question. The authors have chosen a systematic literature review following the PRISMA-P guidelines, which is well-suited for addressing the research question. This methodological approach is appropriate for collecting, synthesizing, and analyzing existing literature to determine how the AECMO&C industry can adapt to Industry 5.0 concepts and technologies to enhance conservation practices for built cultural heritage.

 Sufficient details of the methods are provided to allow replication by others. The protocol outlines specific inclusion and exclusion criteria, the selection of databases, and the search queries used. The systematic and transparent approach ensures that other researchers can replicate the study. The use of PRISMA-P guidelines further strengthens the methodological rigor, ensuring comprehensive reporting and synthesis of findings.

 The datasets are clearly presented in a usable and accessible format. The authors provide links to the underlying and extended data on Zenodo, along with detailed descriptions of the data availability and the terms of the Creative Commons Attribution license. This transparency allows other researchers to access and utilize the datasets for further research or replication studies.

Is the study design appropriate for the research question?

Yes

Is the rationale for, and objectives of, the study clearly described?

Yes

Are sufficient details of the methods provided to allow replication by others?

Yes

Are the datasets clearly presented in a useable and accessible format?

Yes

Reviewer Expertise:

As an expert in multidisciplinary research, my expertise spans artificial intelligence, neurohumanities, neurodiversity, immersive realities, and the gamification of education. With multiple advanced degrees, including a Ph.D. in Artificial Intelligence and extensive experience in academic and administrative positions, I have developed a deep understanding of integrating AI in various fields. My scholarly portfolio includes several books on the application of AI in education and cultural heritage, as well as numerous articles and case studies. This background equips me with a comprehensive perspective on the convergence of technology and humanities, particularly in the context of Industry 5.0 and its implications for conservation practices.

I confirm that I have read this submission and believe that I have an appropriate level of expertise to confirm that it is of an acceptable scientific standard.

# Reviewer report: Reviewer response for version 1

**DOI:** 10.21956/openreseurope.18919.r41174

**Published:** 2024-07-08

**Authors:** Stacy Vallis

**Affiliations:** 1 Auckland University of Technology, Auckland, Auckland, New Zealand

The research presents a timely and valuable proposition concerning how the Architecture, Engineering, Construction, Management, Operation, and Conservation (AECMO&C) industries may adapt to Industry 5.0 concepts. Acknowledging the transition from Industry 4.0 to the Industry 5.0 paradigm, the study is focused on a topic requiring further investigation, being the relationship of the Architecture, Engineering, Construction, Management, Operation, and Conservation (AECMO&C) industry to the new opportunities that are presented. The research protocol presents a comprehensive description of the systematic literature review methodology that has been employed, together with providing a publicly accessible dataset of the bibliographic findings.

 Offering an existing definition for the terms ‘Industry 4.0’ and ‘Industry 5.0’ would be valuable for the reader to understand the key shifts that are taking place. It is helpful to view the current challenges with the existing Industry 4.0 applications to creating digital twins as explained in this research protocol. What are any unique characteristics of Industry 5.0 that are projected to shape workflows in novel ways?

 It would be interesting to also consider how Industry 5.0 technologies can be adapted to address specific needs of the AECMO&C industries e.g. how do the three foundational ideas apply to specific contexts, especially when working with cultural and historic buildings or sites? Could the process of adaptation be a reciprocal one? Additionally, would it be possible to briefly state why the 2020-2024 period is highlighted as a relevant timescale for the systematic literature review? An indication of the main themes for the narrative synthesis of findings might be interesting to provide, to offer a further view towards the subsequent stages of research.

Is the study design appropriate for the research question?

Yes

Is the rationale for, and objectives of, the study clearly described?

Yes

Are sufficient details of the methods provided to allow replication by others?

Yes

Are the datasets clearly presented in a useable and accessible format?

Yes

Reviewer Expertise:

Architecture and emerging technologies for sustainable urban transformation, adaptive reuse and retrofit for historic buildings, heritage documentation

I confirm that I have read this submission and believe that I have an appropriate level of expertise to confirm that it is of an acceptable scientific standard.
